# Experiential learning methods for biostatistics students: A model for embedding student interns in academic health centers

**DOI:** 10.1002/sta4.506

**Published:** 2022-12-25

**Authors:** Gina‐Maria Pomann, L. Ebony Boulware, Cliburn Chan, Steven C. Grambow, Alexandra L. Hanlon, Megan L. Neely, Sarah B. Peskoe, Greg Samsa, Jesse D. Troy, Lexie Zidanyue Yang, Samantha M. Thomas

**Affiliations:** ^1^ Department of Biostatistics and Bioinformatics Duke University Durham North Carolina 27710 USA; ^2^ Duke Clinical and Translational Science Institute Duke University Durham North Carolina 27710 USA; ^3^ Duke Center for AIDS Research Duke University Durham North Carolina 27710 USA; ^4^ Center for Human Systems Immunology Duke University Medical Center Durham North Carolina 27710 USA; ^5^ Center for Biostatistics and Health Data Science, Department of Statistics Virginia Tech Roanoke Virginia 24016 USA; ^6^ Duke Cancer Institute Duke University Durham North Carolina 27710 USA

**Keywords:** collaborative biostatistician, collaboration and consultation, experiential learning, internship model, training biostatisticians

## Abstract

This manuscript describes an experiential learning program for future collaborative biostatisticians (CBs) developed within an academic medical program. The program is a collaborative effort between the Biostatistics, Epidemiology, and Research Design (BERD) Methods Core and the Master of Biostatistics (MB) programme, both housed in the Department of Biostatistics and Bioinformatics at Duke University School of Medicine and supported in partnership with the Duke Clinical and Translational Science Institute. To date, the BERD Core Training and Internship Program (BCTIP) has formally trained over 80 students to work on collaborative teams that are integrated throughout the Duke School of Medicine. This manuscript focuses on the setting for the training program, the experiential learning model on which it is based, the structure of the program, and lessons learned to date.

## 1 INTRODUCTION

Collaborative biostatisticians (CBs) play an essential role in clinical and translational research, contributing to the design, implementation, analysis, and interpretation of research studies (Pomann et al., 2020). The need for CBs continues to grow across the clinical and translational enterprise, and it is critical that trainees are prepared to integrate effectively in this highly collaborative research environment (Begg & Vaughan, 2011; Davidson et al., 2019; Sharp et al., 2021). Academic training for CBs often places more emphasis on the “biostatistics” rather than the “collaborative,” even though both elements are essential to the success of a CB. Academic training on collaboration is often limited to a single course in “consulting,” with or without a supplemental 1‐1 apprenticeship model where students work on a research project with a single faculty advisor (Taplin, 2003). Students typically observe how the advisor navigates the collaborative elements of the project and gradually transition towards taking on some of those responsibilities by playing an increasingly active role in meetings with clinical investigators, proposing analytic strategies, drafting increasingly large sections of manuscripts, and so forth. This traditional approach fails to provide a standard set of educational principles, as the resulting lessons each student learns are largely driven by the specific mentor and research setting at hand. In addition, this 1‐1 apprenticeship model lacks scalability; as the number of students enrolled in graduate programs continues to grow, there are not enough faculty members available to serve as advisors to accommodate a 1‐1 model. An alternative model is critical to maintain consistent training for CBs in graduate programs and ensure growth for larger or expanding programs.

Vance and Smith have proposed a framework for approaching collaboration that emphasizes the importance of developing a relationship between a statistician and the domain expert. Pomann et al. proposed a list of 16 key competencies essential for an effective collaborative biostatistician that fall under three overarching competency pillars: (1) Clinical and Domain Knowledge; (2) Statistical Expertise; and (3) Communication and Leadership (Esterhuizen et al., 2021; Pomann et al., 2020; Spratt et al., 2017; Vance, 2020; Vance & Smith, 2019). While some of these skills can be taught in a traditional didactic setting, experiential training provides an effective way to enhance and reinforce a broader skillset. Experiential training opportunities, such as internships, provide students with firsthand experiences that teach them to develop and navigate relationships with clinical and translational science colleagues. Rapid development of more structured experiential learning opportunities for students will help the field meet the growing demand for highly skilled quantitative staff and faculty CBs. We describe a program that delivers instruction with the above characteristics through a partnership between a graduate program in biostatistics and a collaborative biostatistics group, which provides statistical expertise to biomedical research projects throughout our institution. First, we describe the setting of the program by reviewing the background and rationale. Next, we explain in detail the didactic curriculum and experiential learning model. Third, we present the program timeline and methods for student selection. Last, we discuss the strengths and challenges of this program along with the observed outcomes of the students who have participated over the last 5 years.

### 1.1 Program background and rationale

The Duke University School of Medicine offers training programs in a variety of clinical and basic science specialties. The Department of Biostatistics and Bioinformatics is one of the basic science departments and houses, among other programs, the Master of Biostatistics (MB) and PhD in Biostatistics Programs. The MB Program is a 2‐year professional degree program that trains future CBs and enrolled its first cohort of students in 2011. Approximately 75% of graduates enter the workforce immediately after graduation, with the remaining graduates entering PhD programs. In addition to statistical coursework, MB students take a year‐long sequence in the practice of Biostatistics. This sequence covers topics such as clinical and domain knowledge (e.g., biomedical topics, reading, and interpreting research papers), manuscript development, study design, statistical writing, and presentation skills. The program emphasizes flipped classroom approaches and active learning exercises with a strong focus on real‐world case studies. While this exposes students to the essential components of collaboration, it does not inherently provide them with firsthand collaboration experience. For this reason, all students in the MB program are required to participate in an experiential learning opportunity, a practicum, prior to graduation. This requirement ensures all graduates to leave the program with hands‐on experience working with real data in a collaborative team science setting.

The Biostatistics, Epidemiology, and Research Design (BERD) Methods Core is a group of over 40 staff and faculty biostatisticians who collaborate on interdisciplinary research teams across the institution. The BERD Core, housed within the Department of Biostatistics and Bioinformatics, is supported in part by a National Institutes of Health (NIH) Clinical and Translational Science Award (CTSA) awarded to the Duke Clinical and Translational Science Institute. The Core's main objectives are to connect investigators with appropriate quantitative and qualitative collaborative methodologists, provide staff and faculty CBs for interdisciplinary research projects, and build additional biostatistics resources at the institution as needed. In 2012, the MB program partnered with the BERD Core to initiate the BERD Core Training and Internship Program (BCTIP). BCTIP created a structure to integrate students into collaborative research teams, helping to meet the MB program's need for experiential learning opportunities for its students while also providing biostatistics support to collaborative research teams throughout the institution. Since then, we have accumulated nearly a decade of experience with BCTIP, enrolling over 80 students in this hybrid (i.e., didactic and experiential) training program. During the program, student interns complete formal coursework aimed at developing key competencies (Pomann et al., 2020) and serve as biostatisticians on collaborative research teams. These are collaborative, interdisciplinary teams working on one or more research question/study within a particular domain, embedded throughout the School of Medicine. This model continues to evolve in response to changes in programmatic requirements and collaborative needs.

### 1.2 Didactic training curriculum

BCTIP aims to provide foundational training towards mastering the 16 key competencies for collaborative biostatistics proposed by Pomann et al. (2020).

BCTIP interns work on diverse projects with staff and faculty across many clinical and translational areas. Both didactic and experiential training methods are used to provide coverage of the competencies interns will develop throughout their internship experience. During the first 6 weeks of the training program, interns participate in a didactic course that introduces the fundamental competencies crucial for collaborating with biomedical colleagues (see Appendix S1 for the curriculum and Appendix S2 for expectations). Staff and faculty biostatisticians within the BERD Core lead these training sessions. Topics covered include coding and data manipulation, collaboration and communication, statistical analysis plans, file/folder organization, generation of statistical reports and statistical writing, professionalism, and career preparation. BCTIP interns complete this didactic curriculum concurrently as they begin to work on their assigned projects.

BCTIP enhances the traditional didactic course experience by incorporating the key learning objectives of the internship into course materials and providing real‐world training exercises to highlight these objectives. First, each lecture emphasizes the importance and practical relevance of each topic to their work on assigned projects. For example, students learn how to organize comments and utilize literate programming tools (e.g., RMarkdown and ODS Output) in their statistical code so that their analyses meet reproducibility standards and can be easily reviewed and validated. Second, under the supervision of staff and faculty CBs, students work through training exercises based on their ongoing research projects to build skills in a real‐world setting. These exercises include meeting with collaborators, scoping out the needs of a project, writing statistical analysis plans, performing data analyses, writing summary reports, and reviewing and revising manuscripts. At the conclusion of the didactic training, interns are expected to solidify the skills and information conveyed during training while they gain new skills through experiential learning.

### 1.3 Mentored experiential learning model

The BCTIP experiential learning model incorporates two related but distinct mentoring roles intended to help students work effectively in a dynamic, team science environment. A program advisor oversees administrative aspects of the learning experience (e.g., logistics of supervisor‐student pairings) and a project supervisor oversees the day‐to‐day experiential training experience, is responsible for helping the student develop key collaborative skills, and serves as the biostatistician of record for the intern's assigned collaborative projects. These distinct mentoring roles separate program and project supervision into two complementary roles typically taken on by different biostatisticians.

The BCTIP director and assistant director(s) generally serve as program advisors, overseeing the hiring process, training program, and interactions between the interns and their project supervisors. This includes providing interns with a confidential outlet for discussion of concerns and communication challenges experienced within their collaborative teams or with their project supervisors, supporting conflict resolution as needed. These individuals are experienced practicing biostatisticians whose personal expertise and working experience support program design and mentorship. Program advisors also provide career mentorship including resume review and interview coaching. This dual mentorship model trains the student to collaborate effectively with a team of biostatisticians and clinical/translational researchers while ensuring that the project has oversight from an established biostatistician. Our experience has shown that the separation of roles between program advisors and project supervisors is important because it allows the intern to approach the program advisor when uncertain how to navigate a situation with their project supervisor, for example, how to renegotiate deadlines. Advisors hold quarterly meetings that include all interns in a group discussion about ongoing projects, collaborative issues and questions, internship progress, and career development. This provides interns with a sense of belonging to a community of peers and creates opportunities for peer mentoring.

BCTIP requires each project supervisor to review and sign a memorandum of expectation (MOE) prior to the beginning of the internship. The MOE clearly outlines supervisor expectations and obtains explicit agreement to fulfil these expectations (see Appendix S3). As the biostatistician of record for the intern's assigned collaborative project, each supervisor is responsible for leading the development of the project's statistical analysis plan (SAP), reviewing the intern's work, providing constructive criticism and advice, and discussing relevant statistical approaches and methodology. Supervisors monitor intern work hours and serve as a mentor in the collaborative setting. This may include activities such as reviewing email drafts before sending, discussing potential questions for the study team before team meetings, and providing examples of effective communication strategies during meetings. Project supervisors typically meet weekly with interns so they can provide ongoing mentorship and accurate assessment of the progress and development over the course of the internship.

Every month, BCTIP advisors collect progress reports from each intern (see Appendix S4). They share these reports with the project supervisors only at the discretion of the intern and BCTIP director. Additionally, program advisors collect feedback from project supervisors at the end of each semester and summer session (see Appendix S5). They share constructive performance feedback with the interns at the end of each semester to help them continue to develop their skills. Program advisors meet with project supervisors on an as‐needed basis to discuss challenges and gather training topic suggestions for future cohorts.

### 1.4 Program timeline

**FIGURE 1:**
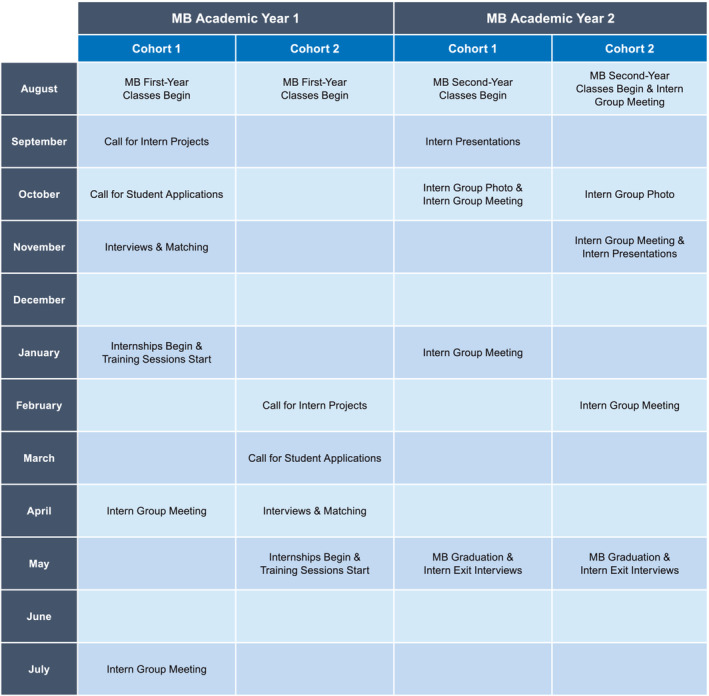
Major components of BCTIP and corresponding timeline

Figure 1 provides an overview of the major components of BCTIP. Two cohorts are hired each year: One during January following completion of the first‐year students' first semester (“Cohort 1”), and one during May following the students' completion of the full first year (“Cohort 2”), with the completion of BCTIP coinciding with graduation from the MB program. Having two cohorts ensures that there is overlap between the current intern and the new intern to prevent any interruption in project support. This overlap also provides an opportunity for the existing intern to familiarize the new intern with project details, workflow dynamics of the collaborative team, and the materials and knowledge needed to successfully replace the current intern on the project. This structure provides seamless support to collaborative teams while allowing the existing intern to gain near‐peer mentoring and leadership experience before graduation. It also provides the opportunity for students to start their internship experience during their first year of study.

Because each of the cohorts enters the program at different points in their graduate training trajectories, the initial 6‐week didactic training curriculum differs with respect to the material covered in the coding and data manipulation training sessions to complement the coursework relevant to each cohort. The other training sessions are consistent for both cohorts. For Cohort 2 interns, BCTIP provides a 2‐week interruption in the training schedule during the summer following the first year of coursework to allow for focused study time for the qualifying exam.

Once the interns have completed at least 6 months of the internship, the program advisors organize a required seminar series that provides interns with the opportunity to practice their scientific communication skills by presenting a summary of their current internship work to an audience of graduate students, staff and faculty biostatisticians, and clinical collaborators. The program advisors and project supervisors assist interns in preparing for these presentations and each intern receives audience feedback after the seminar.

### 1.5 Project matching and student selection

BCTIP hires eight to 20 interns per year (four to 10 interns per cohort), depending on collaborative group needs. When classes are in‐session, interns work 10–20 h per week, and 10–40 h during school breaks. Interns may work on a single long‐term project, several projects within a collaboration, or on several projects across multiple collaborations during their internships (see Appendix S6 for intern project examples). Interns are required to submit monthly progress reports, participate in quarterly intern meetings with BCTIP leadership, and present on their internship work. Intern work schedules vary depending on funding availability and academic hour requirements. Interns are paid at a graduate student rate set up by the university.

Faculty and staff overseeing collaborations that would benefit from student support can submit a formal request to BCTIP for an intern using a survey form (see Appendix S7). The funding source and availability for the intern should be considered by the faculty/staff member before submitting a request. The request period for Cohort 1 is in the fall and for Cohort 2 is in the spring. BCTIP program advisors review all requests and follow up with requesting groups as needed to discuss specific details such as desired skills and characteristics, estimated duration and work schedule, availability of computing resources, and funding information. After the request period has closed, all MB students receive an email notification with details about the internship program and application process. Students must submit a cover letter and resume or curriculum vitae and fill out a short survey (see Appendix S8). Once the application deadline has passed, program advisors review all applications and schedule the initial round of interviews.

Program advisors evaluate all candidates on three core skill domains: Communication, statistical methods, and coding ability using an A/B/C/D/F scale to make the interview process more efficient and equitable. Each applicant proceeds through a first round of interviews with program advisors and is asked the same base set of statistical questions and provided with the same sample code snippet to evaluate. The scoring during the initial interview is used to recommend applicants for secondary interviews and to make hiring decisions. Secondary interviews take place with the specific project supervisors who will be directly overseeing the intern's project work.

Typically, two to three candidates are selected for secondary interviews for every open position, with some interviewing for more than one. This provides students with interview experience and allows individual supervisors to rank their top candidates. Rankings are kept confidential and serve to help program advisors make the best matches based on both student and supervisor preferences. Secondary interviewers often focus interview questions on the key skills desired for the specific project work to identify the best candidate for that position. Hiring decisions are driven by and made after collecting feedback from all secondary interviewers. Formal offer letters are prepared and provided to the selected applicants for signature and return to finalize hiring.

The enrolment size of the MB program has increased over time from its inception in 2011 when 16 students enrolled, increasing to 27 students in 2016 and 48 in 2021. The number of applicants for BCTIP has also increased, with 14 and six applicants for 2017 cohorts 1 and 2, respectively, and 16 and 14 applicants for 2022 cohorts 1 and 2, respectively. The number of hired interns has remained relatively stable with eight and six hired into 2017 cohorts 1 and 2, respectively, and seven and six hired into 2022 cohorts 1 and 2, respectively.

### 1.6 Program outcomes

Since 2017, when we began collecting data on program participants, 65 interns have successfully completed BCTIP and graduated from the MB programs: 24 subsequently entered positions in industry, 23 entered positions in the academic medical field, 14 entered PhD programs, and four obtained positions outside of the United States. Exit surveys have shown that interns feel the program prepared them for the next steps in their careers, with 93% of respondents agreeing with the statement “As a result of completing this internship, I feel competent to work as an entry‐level biostatistician in an academic or industry setting.”

Supervisor feedback indicates that project supervisors see improvement in intern performance over the internship period including development of time management skills, advancement of programming and statistical abilities, and improvement in written and verbal communication. Even so, supervisors have expressed the desire for interns to work more efficiently and complete tasks more quickly. Project supervisors also often collect feedback on intern performance from their clinical collaborators, which is used to guide detailed supervisor feedback to interns and/or used by program advisors to expand the didactic training curriculum.

Program advisors meet with interns quarterly to solicit internship experiences, including suggestions for program improvements and reinforcement of existing program aspects, such as specific training sessions. The internship exit survey also includes open‐ended questions soliciting additional feedback about the program. While most do not provide extensive open‐ended responses, some candid suggestions have spurred program improvements. Program advisors also ask project supervisors to evaluate intern progress at several timepoints throughout the internship. Although most of the feedback collected in this way is intern‐specific and not directed towards the greater program, it has also prompted positive changes in the program. A recent example of how input from interns and project supervisors has been used to improve the program is the career preparation and professionalism training session, which was added to the curriculum based on project supervisor feedback about the need for formal professionalism training and intern feedback about the desire for more focused discussion on resume‐building and interview skills.

## 2 INTERN EXPERIENCE NARRATIVES

This narrative was written by a former MB student and BCTIP intern who graduated from the program in 2018.

After I completed the first year of the master's program at Duke, I participated in BCTIP in the second cohort of 2017. During the one‐year internship, I worked with my project supervisor, a faculty member in the Department of Biostatistics and Bioinformatics and Duke Cancer Institute (DCI), and a clinical collaborator who was an oncologist on a research project to study the toxicity attributed to different courses of chemotherapy/radiotherapy in lung cancer patients. It was the first time I ever worked with real patient data and programmed in SAS. I also learned new statistical methods that were not covered in classes at that time such as the proportional odds cumulative logit model, survival analysis, and imputation for missing data. My project supervisor was very supportive in all aspects, including guiding me through the research collaboration process, teaching me statistical knowledge, and reviewing my work. I also had a chance to interact with a staff statistician in DCI throughout the internship. By observing her daily work, I was able to have a deeper understanding of a master‐level biostatistician's job responsibilities. This truly inspired me to apply for a similar position in academia after graduation, because I wanted to practice the statistical knowledge I learned from coursework while focusing on the mission to improve health outcomes through collaboration with different clinical investigators in the Duke Health system. I joined the BERD Core as a staff biostatistician after graduation and found, not surprisingly, that while a full‐time job requires more independence and competency, the role of a BCTIP intern and that of a full‐time biostatistician within the BERD Core were very much alike. I am grateful that I had the internship experience, which prepared me well as a fresh graduate who felt confident to be qualified for this job and adapt quickly to the working environment.

This narrative was written by a former MB student and BCTIP intern who graduated from the program in 2019:

I began the BCTIP internship during my second semester of the master's program. I worked with my intern supervisor, a faculty member in the School of Nursing, on intensive mobile health data for patients with type II diabetes. I learned how a real biostatistician works and collaborates with medical and nursing researchers in practice. My supervisor patiently guided me through managing real patient data, programming efficiently in both R and SAS, and communicating clearly and effectively with collaborators. It was a great practicum for me to apply statistical concepts learned from coursework as well as build my skillset toward an independent, experienced biostatistician. Moreover, I had a chance to connect with other biostatisticians during my internship and learned a lot from their experiences. Later, we developed the intern project into my master's thesis project after a discussion with my supervisor. These projects exposed me to different phases of research work in biostatistics. I became really interested in exploring new methodology and decided to further pursue a PhD degree in biostatistics. In my current PhD work, I am benefited from the trained research, project management, and programming skills acquired during BCTIP. I also participate in consulting projects as a PhD student, and the BCTIP experience prepared me to work closely with collaborators. I really appreciate the opportunity to work as a BCTIP intern not only for the experience with technical research projects, but also for the mentorship, support, and feedback from my supervisor and other biostatisticians. In BCTIP, I learned what a successful biostatistician and researcher should be like.

## 3 DISCUSSION

BCTIP provides students with valuable professional experience that distinguishes them in the eyes of hiring managers and PhD program selection committees. BCTIP alumni demonstrate many of the communication and project management skills that can only be gained through real‐world project experience alongside solid training in statistical methodology. Students who complete BCTIP have been very successful at securing full‐time statistician positions across industry and academia, as well as gaining acceptance to PhD programs. Although a formal study has not been conducted comparing post‐graduation outcomes in students who did and did not participate in BCTIP, anecdotally, feedback from hiring managers who have hired or worked with BCTIP graduates suggests they regularly seek out BCTIP alumni for new positions. Future studies comparing these student groups are warranted.

The benefits of BCTIP are apparent not only to program and department leadership but also to MB students. Each fall and spring, when the BCTIP leadership team is preparing to release the call for intern applications, there is overwhelming interest from the MB student body and the program teaching faculty. BCTIP leaders are regularly asked to sit on internship panels for the MB program and serve as resources for faculty and staff statisticians who have questions about internships and student research experiences.

One of the primary strengths of BCTIP is how it efficiently integrates interns into collaborative teams across Duke University, an institution with many collaborative teams that include biostatisticians. The strong demand for student support in the BERD Core has resulted in increasing student involvement in these collaborations. Students are strongly interested in these experiential training opportunities, especially because the MB program requires that students complete a practicum and a master's project/thesis. Interns do not receive course credit for participation in the internship, but in many cases, much of the BCTIP intern's project work can be used towards the completion of their required practicum or master's project.

Program and departmental leadership have learned valuable lessons throughout the development of BCTIP. The tight integration between programs has required BCTIP and MB program leadership teams to work closely together, deciding which topics are best included as part of formal coursework and which are supplemental and better included as part of BCTIP training sessions. Students in the MB program can select one of three different program tracks (clinical and translational research, mathematical statistics, or biomedical data science), adding additional complexity and considerations to the core content that should be considered base knowledge taught as part of regular coursework.

This close collaboration has led to mutually beneficial innovations, thereby contributing to the success of each program. For example, the instructional approaches developed in BCTIP along with the robust continuous assessment components of the program have helped inform the redesign of the MB program's first‐semester coursework in the practice of collaborative biostatistics. Similar continuous assessment approaches used in the MB program have led to reciprocal program improvements in BCTIP.

BCTIP is not without challenges and limitations. The internship is only available to students in the MB program at Duke University. The number of students enrolled in the MB program has increased over time, resulting in an increase in the number of applicants to BCTIP; however, supervisory effort and project availability pose barriers to increasing the number of interns in BCTIP at a similar rate. The number and variety of internship projects are reliant on and coordinated by faculty and staff within the department of Biostatistics and Bioinformatics. Securing funding for an intern from a collaborative team often requires substantial time and coordination.

Each student and corresponding project supervisor have project‐appropriate effort funded by the clinical/translational research funding corresponding to a specific project. Each funding source needs to be verified and business management support must be available to help manage the back‐end finances of the program. To support sufficient time for program advisors, BCTIP must cover approximately 25%–40% effort for established biostatisticians. The program also utilizes volunteers to teach the didactic training curriculum; if volunteers are not available, additional funding is required to provide salary support for teaching efforts in the program.

The whole process of hiring students is also a substantial undertaking for the human resources department. An additional challenge is identifying available funding to support and maintain both the student positions and the dedicated administrative time required to oversee these internship experiences. Increasing effort for program leadership and administrative support would allow for additional student intern collaborative opportunities, so it is essential to consider how funding will be secured for these activities.

Implementation of BCTIP in the Department of Biostatistics and Bioinformatics at Duke University School of Medicine has benefited many MB students and ongoing research collaborations across the institution. We have successfully trained and graduated over 80 students during 9 years and have seen impressive growth in both the breadth of the didactic training curriculum and the careful oversight of the experiential internship. We hope that our experience will benefit other institutions seeking to develop and implement internship training program targeted at preparing their students to be effective collaborative biostatisticians. We hope to continue growing and evolving this program to include more collaborative opportunities for students, while maintaining scientific and statistical rigor.

## CONFLICT OF INTEREST

SCG reports receiving consulting fees from Gilead Sciences for serving on multiple Data Monitoring Committees. Although this relationship is not perceived to represent a conflict with the present work, it has been included in the spirit of full disclosure. There are no other conflicts of interest to report.

## Supporting information


**Appendix S1.** Training Curriculum
**Appendix S2.** Intern Expectations
**Appendix S3.** Memorandum of Expectation (MOE)
**Appendix S4.** Monthly Progress Report
**Appendix S5.** Supervisor Feedback Survey
**Appendix S6.** Intern Project Examples
**Appendix S7.** Call for Intern Support Survey
**Appendix S8.** Applicant Survey
**Appendix S9.** MB Program

## Data Availability

Data sharing not applicable to this article as no datasets were generated or analyzed during the current study.
