# Supplementary material for: Experiential learning methods for biostatistics students: A model for embedding student interns in academic health centers
Source: Stat. 2022 Dec 25;11(1):e506. doi: 10.1002/sta4.506 (PMC10022448; doi:10.1002/sta4.506)
Supplement: Supplementary file 1 — Appendix S1. Training Curriculum Appendix S2. Intern Expectations Appendix S3. Memorandum of Expectation (MOE) Appendix S4. Monthly Progress Report Appendix S5. Supervisor Feedback Survey Appendix S6. Intern Project Examples Appendix S7. Call for Intern Support Survey Appendix S8. Applicant Survey Appendix S9. MB Program [file STA4-11-0-s001.docx]

**Supplemental Material**

**Experiential Learning Methods for Biostatistics Students: A Model for Embedding Student Interns in Academic Health Centers**

**Appendix A. Training Curriculum**

Week 1: Introduction and Orientation

This 2-hour session introduces the internship program and the BERD Core to new hires. Interns are informed of their primary responsibilities, expectations, and requirements, as well as the technical support and statistical resources available in the BERD Core. Program leadership are available after the presentation to answer questions, help with computer set up, etc.

Week 2: Collaboration and Communication

This 1-hour session covers topics related to collaboration and communication commonly encountered in the Duke collaborative research setting, such as the distinction between collaboration and consultation, the BERD Core collaborative model, professional communication (written and verbal), Rindskopf’s and Grambow’s rules of communication, and strategies for dealing with mistakes.

Week 2-6: Coding and Data Manipulation

R and SAS software platforms are introduced in a total of 4 2-part sessions given over 5 weeks. These sessions are designed to complement the software courses included in the MB program curriculum. Each software platform is introduced at a basic level first and then extended to a more advanced level. Part 1 of each session is an introduction, which includes an assignment activity, followed by part 2 the following week, which serves as an assignment review session. These sessions cover a variety of topics, including frequently used commands/procedures, efficient coding skills, programming best practices, tables and plots, and functions/macros.

Week 3: SAPs and Project Management

As a biostatistician, good project management skills are vital in order to achieve data transparency, traceability, and reproducibility. This 1-hour session covers two key aspects of project management: statistical analysis plan (SAP) development and file organization/documentation. The purpose and process of writing SAPs are discussed with templates and examples provided. Additionally, readme files, efficient ways of file organization, and file naming conventions are introduced with examples.

Week 3: Professionalism and Career Preparation

This 1-hour session focuses on essential working skills such as professional attire, interview skills, leading meetings, and verbal and written communication. Topics discussed include intern expectations and soft skills that are important in biostatistics collaborations including time management, networking, teamwork, creative thinking, and conflict resolution.

Week 6: Reporting and Statistical Sections

The importance and process of writing analysis reports and statistical sections in manuscripts are introduced in this 1-hour session. Components of reporting in the BRED Core, reporting standards, and guidelines for scientific journals are addressed with examples, followed by a short team practice and discussion.

**Appendix B. Intern Expectations**

**Work Schedule**

- Communicate with supervisor about expected number hours of work per week.
- Provide a fixed schedule with location so that the supervisors can find or contact them if needed.
- Communicate with supervisor about time off. Notify supervisor as soon as possible of any plans for time off. Give at least one-month advance notice if the intern intends to take off a week or more at once.
- Inform supervisor if the intern is not able to work the fixed schedule well in advance. This should not happen often and should only be for extenuating circumstances. As a reminder, in order to comply with the student work policy of no more than a total of 19.9 worked hours per week, make up time should always be worked in the same Monday to Sunday 7-day period and not in the week before or after the change in schedule.
- Discuss with supervisor if the intern would like to increase or decrease number of work hours.
- At the end of each week (usually Friday), the intern is expected to send their supervisor a task list with projected time and actual time for each task and their accomplishments for the past week.

Example of task list

**Week 6/6 – 6/10** (Note: current week)

| Task | Supervisor | Status | Note | Projected time | Actual time |
| --- | --- | --- | --- | --- | --- |
| Programmed descriptive statistics on data set | Staff/Faculty | Complete | Code sent to Supervisor on 6/8 | 2-2.5 hours | 2.5 hours |
| Created baseline table for subset dataset | Staff/Faculty | Complete | Table in excel file named subset_baseline_tab.xlsx | 1.5-2 hours | 1.5 hours |
| Rewrite code for calculating outcomes (length of stay, multiple diagnosis, comorbidity score, cost) | Staff/Faculty | In progress | Waiting for Supervisor to explain how to calculate comorbidity score to complete task | 4-5 hours | 3 hours |

**Week 6/13 – 6/17** (Note: next week)

| Task | Supervisor | Status | Note | Projected time | Actual time |
| --- | --- | --- | --- | --- | --- |
| Create demographic table | Staff/Faculty |  |  | 1-1.5 hours |  |
| Run regression analysis outlined in SAP | Staff/Faculty |  | Data will be ready on 6/14 from REDCap. | 4 hours |  |

**Communication**

- Let the supervisor know what they want to achieve during the internship by setting goals with the supervisor at the beginning of each semester.
- Set up a recurring weekly meeting with the supervisor (at least biweekly). Meetings can be scheduled more often if needed.
- Always reply to e-mails the same day or at most within 24 hours (excluding weekends).
- Always send summary/follow-up e-mails after each meeting with supervisor.
- Communicate with investigators independently, professionally, and effectively while copying the correct supervisors on all relevant emails. Always discuss with supervisors prior to sending emails to investigators.
- Write effectively – read other similar papers and mimic the style (DO NOT plagiarize).
- Maintain good time management to meet deadlines and ask for help if the intern cannot complete a task/project.
- Inform supervisor promptly if the intern cannot meet a deadline.

**Evaluations**

- Complete and submit monthly progress report by the 10^th^ of the month to program advisors.
- The supervisor and intern should set aside meeting times at the end of each month to discuss progress and performance. The intern can ask for feedback at any time.
- Provide feedback to the supervisor directly or through the BCTIP advisors. Fill out a self-evaluation form at the end of each semester. The intern will be evaluated based on their goals set at the beginning of each semester.
- The supervisor and intern will have an exit interview at the end of the internship.
- Can request a letter of recommendation from the supervisor or program advisors.

**Skills**

- Master the materials and concepts that have been taught in their MB programming course, BCTIP training sessions, coursework, and previous work.
- Attend all training sessions and submit all assignments.
- Follow the guidelines in this document and other standard operating procedure documents.
- Take the initiative to tackle problems and attempt to find solutions independently. Ask for help from supervisor or another appropriate statistician if a problem is taking too long to solve. Be able to write first drafts of statistical analysis plans that need minimal editing.
- Write reproducible and easy to follow code.
- Be an efficient programmer and be able to work through problems with limited guidance.
- Working knowledge of R and SAS.
- Ability to work independently with proper guidance. Willingness to learn new methods/techniques/ways of doing things.
- Ask for training materials when necessary (e.g. book or online training course). Be familiar with Staff Training folder on BERD Core shared drive.
- Give a presentation on a current project.

**Administrative**

- Submit a list of tasks and how many hours spent on each task at the end of pay period (“time-tracker”).
- Submit timecard(s) by Sunday @ 11:59pm following end of pay period and inform supervisor(s).
- Attend all quarterly intern meetings.
- Discuss internship with supervisor at least 1 month prior to potential end date to confirm continuation.

**Appendix C. Memorandum of Expectation (MOE)**

**Memorandum of Expectation**

**BERD Core Training & Internship Program**

[*Insert Faculty/Staff Supervisor Name*] in the Department of Biostatistics and Bioinformatics (B&B) has agreed to the following terms for a statistical collaboration and intern support beginning on [*Insert Start Date*]. The B&B supervisor, [*Insert B&B Faculty/Staff Supervisor Name*], will supervise an intern as part of the Biostatistics, Epidemiology, Research, and Design (BERD) Methods Core Training & Internship Program (BCTIP) for collaborations within [*Insert Collaborative Group/Department Name*].

A separate Memorandum of Understanding (MOU) will be signed between [*Insert Collaborative Group/Department Name*] and B&B to secure funding for the intern, if applicable. The intern will be employed up to 19.9 hours/week when classes are in session and up to 39.9 hours/week otherwise. A minimum of 4 guaranteed hours of work per week must be provided to the intern for the duration of the internship. The B&B intern statistician will provide statistical support for multiple aspects of the research project. This will include manuscript preparation, general statistical collaboration, and data quality processing.

The intern will participate in the BERD Core Training & Internship Program (BCTIP). As part of this program, the staff statisticians in the Core will provide the intern with professional mentorship. The intern will also have access to the Core’s shared statistical resources and be required to attend quarterly BCTIP intern meetings. Additionally, the intern will participate in up to 30 hours of training and will be compensated for the time spent in training and quarterly BCTIP meetings by [*Insert Collaborative Group/Department Name*]. The collaborative group/department will be charged a fee of [*Insert Fee Amount*] for operational costs, including but not limited to computing and professional development, for each intern that participates in the program. Please select the appropriate option and provide the requested information:

Funding and fees will be paid as outlined in MOU #[*Insert Appropriate MOU #*] between the BERD Core and [*Insert Collaborative Group/Department Name*] which outlines the funding sources for this internship.

Funding for this internship is not included in a current MOU. The following cost object(s) will be used to allocate the effort and fees for this internship:

Intern Effort Fund Code(s): [*Insert Fund Code(s) for Effort*]

Other Fees Fund Code: [*Insert Fund Code for Fees*]

Changes to these cost object(s) will be provided by [*Insert B&B Faculty/Staff Supervisor Name*] to [*Insert BCTIP Contact Name*] no later than 45 days after the effective date of change.

[*Insert B&B Faculty/Staff Supervisor Name*] will provide direct supervision and time card approval for the intern. This includes providing guidance and advice on all aspects of the research project. This supervisor will check in with the student via email or in face to face meetings a minimum of once per week for the duration of the collaboration. The faculty/staff will also provide feedback to the BERD Core regarding the intern’s progress each semester. The intern will present his/her collaboration work during the fall semester. The supervisor is highly encouraged to attend the presentation given by the intern. The purpose of BCTIP is to provide a statistical community and training program for the intern; BCTIP does not provide supervision on research projects.

This agreement will be effective [*Insert Start Date*] through [*Insert Sprint Semester End Date*], and contingent on funding and performance, will be extended through the summer (Ending Date [*Insert Summer Period End* Date]), the fall semester ([*Insert Fall Semester End Date*]), and the spring semester ([*Insert Sprint Semester End Date*]) at the discretion of [*Insert B&B Faculty/Staff Supervisor Name*] without necessity of signature of an additional agreement.

The direct supervisor, [*Insert B&B Faculty/Staff Supervisor Name*], may designate other staff/faculty to provide supervision of the intern. These individuals must be approved by BCTIP and will also be required to sign this agreement. Please list any designated staff/faculty supervisors here:

BY: _______________________________________________________DATE:_________________________________

*B&B Faculty/Staff Supervisor*

BY: _______________________________________________________DATE:_________________________________

Designated Staff/Faculty

BY: _______________________________________________________DATE:_________________________________

Director of BERD Core

cc: BCTIP Leadership Team

 Department Business Manager

**Appendix D. Monthly Progress Report**

**BERD Core Training and Internship Program**

**Progress Report – To Be Completed Monthly**

Name: Date:

Collaboration(s) & Supervisor(s):

Summary of Current Projects:

Progress since Last Report:

What things, if any, prevented or delayed you from achieving your goals for this month?

How (phone/email/meeting) and how often did you contact your supervisor(s)?

Have you verified your fund code(s) with your supervisor who approves your time card(s)? Are there any issues related to the fund code(s) or time card(s)?

Goals for Next Month:

Additional Comments, Concerns, or Complaints:

**Appendix E. Supervisor Feedback Survey**

**BCTIP Supervisor Evaluation**

**Please complete the following evaluation form for any interns you are working with through the BERD Core Training and Internship Program (BCTIP).**

Date

________________________________________________________________

Name of Supervising Faculty/Staff

________________________________________________________________

Name of Intern

________________________________________________________________

Collaborative Group(s)

________________________________________________________________

How often do you meet/communicate with the intern (phone/in-person/virtually/email)?

________________________________________________________________

**Please rate the intern’s performance on the following factors and provide more information.**

*Programming Skills*

| Needs Improvement | Successful | Exceptional | N/A |
| --- | --- | --- | --- |
| 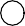 | 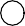 | 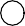 | 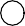 |

Please elaborate:

________________________________________________________________

*Verbal Communication Skills*

| Needs Improvement | Successful | Exceptional | N/A |
| --- | --- | --- | --- |
| 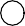 | 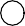 | 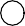 | 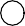 |

Please elaborate:

________________________________________________________________

*Written Communication Skills*

| Needs Improvement | Successful | Exceptional | N/A |
| --- | --- | --- | --- |
| 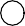 | 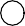 | 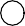 | 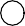 |

Please elaborate:

________________________________________________________________

*Statistical Knowledge*

| Needs Improvement | Successful | Exceptional | N/A |
| --- | --- | --- | --- |
| 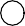 | 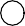 | 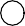 | 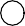 |

Please elaborate:

________________________________________________________________

*Time Management*

| Needs Improvement | Successful | Exceptional | N/A |
| --- | --- | --- | --- |
| 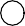 | 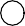 | 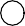 | 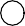 |

Please elaborate:

________________________________________________________________

*Ability to Think Critically about Project*

| Needs Improvement | Successful | Exceptional | N/A |
| --- | --- | --- | --- |
| 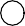 | 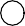 | 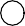 | 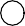 |

Please elaborate:

________________________________________________________________

*Ability to Work Independently*

| Needs Improvement | Successful | Exceptional | N/A |
| --- | --- | --- | --- |
| 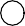 | 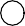 | 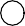 | 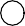 |

Please elaborate:

________________________________________________________________

Has the intern shown improvement? Please explain.

________________________________________________________________

Additional Comments

________________________________________________________________

**Appendix F. Intern Project Examples**

| **Research Question** | **Statistical Skills Needed** | **Description** |
| --- | --- | --- |
| Is 21-Gene Recurrence Score Associated with Axillary Nodal Disease Burden? | Basic data cleaning and manipulation; utilization of large administrative database; unadjusted parametric (chi-square, ANOVA, t-test) and non-parametric (Wilcoxon rank sum, Wilcoxon signed rank, Kruskal-Wallis) testing; logistic regression; generalized estimating equations. | Intern reviewed code and analysis reports from a previous similar project and researched logistic regression and generalized estimating equations to gain required skills. Intern worked through statistical questions and issues with the project supervisor. The project resulted in a conference presentation and publication. |
| Does MLH1 hypermethylation predict poor outcomes with immune checkpoint inhibitors in recurrent endometrial cancer? | Intermediate data cleaning and manipulation; utilization of prospectively-collected multi-site study data; unadjusted parametric (chi-square, ANOVA, t-test) and non-parametric (Wilcoxon rank sum, Wilcoxon signed rank, Kruskal-Wallis) testing; logistic regression; unadjusted survival analysis (Kaplan-Meier). | Intern reviewed code and analysis reports from a similar project and researched logistic regression and survival analysis to gain the required skills. Data were extremely disorganized and needed a good amount of cleaning and manipulation – the intern was able to get data into a suitable format for analysis. The intern worked through statistical questions and issues with the project supervisor. The project resulted in a conference presentation and publication. |
| Are there differences in Pulmonary Embolism and Deep Venous Thrombosis rates after Primary vs Revisional Bariatric Surgery? | Basic data cleaning and manipulation; utilization of large administrative database; unadjusted parametric (chi-square, ANOVA, t-test) and non-parametric (Wilcoxon rank sum, Wilcoxon signed rank, Kruskal-Wallis) testing; propensity score matching; logistic regression; modified Poisson regression. | Intern reviewed related code and analysis reports and researched logistic regression, modified Poisson regression, and causal inference methods to gain required skills. Intern worked through statistical questions and issues with the project supervisor, including evaluating multiple modeling approaches and choosing the best option. The project resulted in a publication. |

**Appendix G. Call for Intern Support Survey**

**Call for Intern Support – *[insert cohort]***

**Complete the following form to request intern support through the BERD Core Training and Internship Program (BCTIP).**

**BCTIP will interview and recommend an intern for your position. A Memorandum of Expectation (MOE) will be signed by the collaborative group/department, B&B supervising faculty/staff, and BCTIP outlining the requirements of the B&B supervising faculty/staff and the intern within BCTIP.**

**All interns will have the same start date: *[insert start date]***

***The deadline to submit requests is [insert submission deadline]***.

Please note that per hourly-pay requirements, the intern must be residing in the state of North Carolina to be eligible for hiring.

Each intern will take part in training sessions, quarterly intern meetings, and give a presentation of their project(s) as part of BCTIP, which will account for up to 30 hours of time funded by the collaborative group/department. The collaborative group/department will be charged a fee of *[insert fee amount]* for computing and professional development costs for each intern that participates in the program. BCTIP will not provide statistical support for the intern; that will be left up to the B&B supervising faculty/staff. If support for the intern is not included in a current MOU with the Biostatistics, Epidemiology and Research Design (BERD) Methods Core, an agreement will be generated by the Core.

Please Review the MOE and ensure understanding before proceeding.

Name of Supervising Faculty/Staff

________________________________________________________________

Contact Email Address of the Supervising Faculty/Staff

________________________________________________________________

Name of the PI(s)

________________________________________________________________

Contact Email Address of the PI(s)

________________________________________________________________

How will the intern be hired?

(NOTE: It is recommended that the department that owns the fund code does the hiring)

- Through the Collaborative Group/Department (Recommended)
- Through Biostatistics & Bioinformatics

Please note that BCTIP requires submission of the fund code for the intern at least 2 weeks prior to the hire date to allow adequate time for processing and finalizing the intern position. If a fund code is not received by this time, BCTIP may terminate the internship agreement.

Name of the Financial Contact

________________________________________________________________

Email Address of the Financial Contact

________________________________________________________________

Collaborative Group/Department

________________________________________________________________

Maximum Number of Hours Needed per Week - Summer

(NOTE: Interns may work up to 19.9 hours while classes are in session and up to 39.9 hours during school breaks, contingent on approval from collaborative group/department and faculty supervisor, but must be provided with a minimum of 4 hours of work per week)

________________________________________________________________

Maximum Number of Hours Needed per Week - School Year

(NOTE: Interns may work up to 19.9 hours while classes are in session and up to 39.9 hours during school breaks, contingent on approval from collaborative group/department and faculty/staff supervisor, but must be provided with a minimum of 4 hours of work per week)

________________________________________________________________

Title/Description/Goals of Project(s) - Please be as specific as possible.

_____________________________________________________________________________________________________________________________

_____________________________________________________________________________________________________________________________

What skills and knowledge would the ideal intern possess for this position?

_____________________________________________________________________________________________________________________________

_____________________________________________________________________________________________________________________________

Unless specific criteria are met, BCTIP interns are required to use a Duke-issued computer for all internship work, and all internship-related materials must be stored on a secure and backed-up network folder owned by BCTIP and/or the collaborative group/department.

Computing and Office Needs: Which of the following will apply?

- Intern will be provided with a computer and work space by collaborative group/department
- Intern will require use of a BCTIP-owned laptop and work space
- Other, please explain ________________________________________________

Thank you for your interest in hiring an intern through BCTIP! We will get back to you soon in response to your request. Please email *[insert program leadership]* with any additional questions.

**Appendix H. Applicant Survey**

BCTIP Application - *[Insert Cohort]*

**Complete the following form to apply for an intern position through the BERD Core Training and Internship Program (BCTIP).**

BCTIP will provide a statistical community and training program for the selected Master of Biostatistics student interns. The Biostatistics, Epidemiology, and Research Design (BERD) Methods Core collaborates on a variety of projects that span across the School of Medicine. As a BCTIP intern, you will be matched with one or more projects given your interests and background, and the selection of projects in need of support. You will also have access to the Core’s shared statistical resources and take part in specially designed training sessions. As part of this program, the staff/faculty biostatisticians associated with the BERD Core will provide you with professional mentorship and you will be invited to present at a special seminar as part of the Core’s monthly applied seminars.

BCTIP follows a competitive hiring process where all applicants are required to go through a series of interviews before hiring decisions are made.

All internships will begin on [*insert start date*]. (Exceptions may apply)

**Job Performance Expectations**

- Ability to work in a task and deadline driven environment with often shifting priorities.
- Assurance of maintaining confidentiality of all work.
- Demonstration of initiative and responsiveness.
- Ability to work both independently and collaboratively
- Provide at least one week notice of when normal hours will vary for such things as exams, etc.
- ​​Handle multiple concurrent projects and meet deadlines

**Preferred Skills**

- Strong written and verbal communication
- Strong theoretical background
- Ability to use SAS and R
- Knowledge of basic statistical analysis methodology (power calculations, linear regression, etc.)

Name of the Applicant

________________________________________________________________

How many hours per week do you plan to work during the summer/winter breaks and during the school year?


(NOTE: Interns may work up to 19.9 hours while classes are in session and up to 39.9 hours during school breaks, contingent on approval from collaborative group/department and faculty/staff supervisor, but must be provided with a minimum of 4 hours of work per week)

________________________________________________________________

Please upload your resume.

Please upload your cover letter. In your cover letter, please list any other commitments or positions you currently have or anticipate having and what you hope to gain if you are offered a position with BCTIP.

Thank you for your interest in applying for an intern position through BCTIP! We will get back to you soon in response to your application. Please email *[insert program contact]* with any additional questions.

**Appendix I: MB Program**

The MB program is organized around the "ABCs of biostatistics":

- **Analytical** skills include mastery of statistical analysis methods, statistical programming, and also more general skills in problem definition and problem solving.
- **Biology** skills include a combination of subject matter proficiency in biology and medicine focused on the ability to integrate biological concepts into research study design and data analysis, with an emphasis on strategies for learning scientific content as needed.
- **Communication** and team science skills include taking in information from outside the discipline, explaining and advocating for appropriate statistical methods, and effectively participating on teams with scientists across biology and medicine.

To achieve this mission, we have developed a fast-paced, rigorous 2-year graduate program. In their first year, students take courses in mathematical statistics, applied statistical methods (i.e., competency A); statistical programming (i.e., competency B); and statistical practice (i.e., competency C).  In their second year, students select electives consistent with an emphasis on mathematical statistics, biomedical data science, or clinical and translational science. They also complete a master's project, which is intended to integrate the above ABCs. Internships, research projects, and other opportunities to engage in supervised practice of biostatistics are encouraged. A distinctive feature of the program is the emphasis on team science, including communication and strategies for attaining sufficient proficiency in scientific content.

The MB program and the BCTIP most closely interact through an internship program developed by the latter and described in the body of this manuscript, which provides valuable experience in serving, under supervision, as a biostatistician within ongoing team science projects. From the perspective of the MB program, being plugged into an existing and thoughtfully designed mentoring structure is especially helpful.
